# PARP inhibitors in metastatic prostate cancer

**DOI:** 10.3389/fonc.2023.1159557

**Published:** 2023-04-24

**Authors:** Amy K. Taylor, David Kosoff, Hamid Emamekhoo, Joshua M. Lang, Christos E. Kyriakopoulos

**Affiliations:** ^1^ Department of Medicine, University of Wisconsin, Madison, WI, United States; ^2^ University of Wisconsin Carbone Cancer Center, Madison, WI, United States

**Keywords:** PARP inhibitor, prostate cancer, DNA damage repair genes, BRCA, homologous recombination repair

## Abstract

Poly-ADP ribose polymerase inhibitors (PARPi) are an emerging therapeutic option for the treatment of prostate cancer. Their primary mechanism of action is *via* induction of synthetic lethality in cells with underlying deficiencies in homologous recombination repair (HRR). In men with metastatic castrate-resistant prostate cancer (mCRPC) and select HRR pathway alterations, PARPi treatment has been shown to induce objective tumor responses as well as improve progression free and overall survival. Presently, there are two PARPi, olaparib and rucaparib, that are FDA approved in the treatment of mCRPC. Ongoing research is focused on identifying which HRR alterations are best suited to predict response to PARPi so that these therapies can be most effectively utilized in the clinic. While resistance to PARPi remains a concern, combination therapies may represent a mechanism to overcome or delay resistance.

## 1 Introduction

Prostate cancer is the most common cancer and the second-leading cause of cancer related mortality in US men, with an estimated 34,700 deaths annually, representing nearly 11% of all cancer deaths in men (1). The incidence rate of prostate cancer has risen by 3% per year from 2014 through 2019; approximately 288,300 men are newly diagnosed with prostate cancer annually (1). The incidence of men presenting with incurable, metastatic disease at the time of first diagnosis has increased from 3% to 8% between 2008 to 2018 (2–4) and appears to be a primary driver in the increased overall incidence of prostate cancer (1). Although the mainstay of treatment is androgen deprivation therapy (ADT), resistance to ADT is a key event in the management of prostate cancer (5). The onset of metastatic castration resistant prostate cancer (mCRPC) is associated with a poor prognosis and worse overall survival (6, 7). Poly-ADP ribose polymerase inhibitors (PARPi) have emerged as an available treatment for a subset of patients with mCRPC (8, 9) (**Tables 1**, **2**). 

**Table 1:** Completed trials of PARPi monotherapy in mCRPC.

Clinical Trial	NCT#	PARPi Studied	Phase	HRR Selection Status	Prior line(s) of therapy required	Outcome of Primary Endpoint	Reference
TOPARP-A	01682772	Olaparib	II	Unselected	ARSI, chemotherapy	88% composite RR in HRR+	(8)
TOPARP-B	01682772	Olaparib	II	HRR+	ARSI, chemotherapy	400mg dose cohort: 54% composite RR 300mg dose cohort: 39% composite RR	(10)
PROfound	02987543	Olaparib	III	Cohort A: *BRCA1/2, ATM* Cohort B: other HRR+	ARSI	Cohort A: rPFS improved 7.4 months (olaparib) vs 3.6 months (ARSI), HR 0.34	(11)
TRITON2	02952534	Rucaparib	II	*BRCA1/2*	ARSI x2, chemotherapy	ORR 43.5%	(9)
GALAHAD	02854436	Niraparib	II	HRR+	ARSI, chemotherapy	*BRCA1/2*: ORR 34.2% Non-BRCA: ORR 10.6%	(12)
TALAPRO-1	03148795	Talazoparib	II	HRR+	ARSI, chemotherapy	ORR 29.8%	(13)

NCT (clinicaltrials.gov registration), Androgen receptor signaling inhibitor (ARSI), homologous recombination repair (HRR), homologous recombination repair deficient (HRR+), response rate (RR), overall response rate (ORR), radiographic progression free survival (rPFS), hazard ratio (HR).

**Table 2:** Selected completed trials of PARPi combination therapies in mCRPC.

Combination Class	Trial, NCT#	PARPi	Combined Agent	Phase	HRR Selection Status	Primary Outcome(s)	Reference
Androgen Signaling Inhibitor (ARSI) therapy	NCT01972217	Olaparib	Abiraterone	II	Unselected	Median rPFS 13.8 mo vs 8.2 mo, HR 0.65	(14)
	NCT03732820 (PROpel)	Olaparib	Abiraterone	III	Unselected	Median rPFS 24.8 mo vs 16.6 mo, HR 0.66	(15, 16)
	NCT03748641 (MAGNITUDE)	Niraparib	Abiraterone	III	A: Unselected B: HRR+	A: No improvement in rPFS, HR 1.09 B: Improved rPFS 16.5 mo vs 13.7mo, HR 0.73	(17, 18)
	NCT01576172 (NCI 9102)	Veliparib	Abiraterone	II	Unselected	PSA_50_ 72% vs 64%	(19)
Immunotherapy	NCT02484404	Olaparib	Durvalumab	II	Unselected	Median rPFS 16.1 mo, PSA_50_ 53%	(20)
	NCT03338790 (CheckMate9KD)	Rucaparib	Nivolumab	II	A: HRR- B: HRR+	A: 14% PSA_50_ RR B: 42% PSA_50_ RR	(21, 22)
	NCT02861573 (KEYNOTE-365)	Olaparib	Pembrolizumab	Ib/II	Unselected	15% PSA_50_ RR; 8.5% ORR	(23)
	NCT03330405 (JAVELIN PARP)	Talazoparib	Avelumab	Ib/II	Unselected	mCRPC, HRR+ cohort: ORR 11.1%	(24)
Chemotherapy	NCT01085422	Veliparib	Temozolomide	II	Unselected	8% PSA_30_ RR	(25)
Targeted Therapy	NCT03787680 (TRAP trial)	Olaparib	Ceralasterib	II	A: HRR- B: *BRCA1/2, ATM*	A: 11% PSA_50_ RR B: 33% PSA_50_ RR	(26)

PARPi act *via* synthetic lethality, a mechanism which exploits an underlying feature such as a genetic mutation to induce toxicity when combined with another agent or genetic mutation (27, 28). In the case of PARPi, synthetic lethality is mediated *via* disruption of the DNA damage response that is essential for maintenance of DNA integrity (29). There have been 17 PARP members identified, however the two predominant enzymes involved in this damage response are PARP1 and PARP2 (30). PARP1 facilitates DNA repair by binding to DNA breaks and forming PAR chains (PARylation) that act as docking sites for DNA repair proteins to act at the site of identified DNA damage (31). PARP1 eventually PARylates itself (autoPARylation) and this likely causes PARP1 to release from repaired DNA (29). In the absence of PARP1, spontaneous single strand breaks have been shown to lead to collapse of the replication fork, which triggers the homologous recombination repair mechanism (31). Other studies have demonstrated that PARPi additionally trap PARP enzymes on the DNA strand by inhibiting normal auto-PARylation (32). When PARP complexes are trapped on DNA, replication is stalled, and an accumulation of DNA breaks has been observed (32). When PARPi are combined with an additional cytotoxic therapy or utilized in the presence of an underlying deficiency in DNA damage repair, it is hypothesized that DNA damage accumulates and ultimately leads to cell death.

Investigation into the use of PARPi lead to the discovery that cells deficient in homologous recombination repair mechanisms are particularly susceptible to the lethality induced by PARPi (31, 33). *BRCA1/2* deficient cells were the first among the homologous recombination deficient phenotypes to be studied with PARPi. In the setting of PARP inhibition, homologous recombination repair is triggered (31). Homologous recombination is a conserved method of DNA damage repair that is relatively error-free (34). However, in the setting of *BRCA1/2* deficiency, the cell becomes reliant upon DNA repair strategies such as non-homologous end joining (NHEJ). Unlike homologous recombination, NHEJ is prone to error as this mechanism directly joins broken ends of DNA with little regard for sequence homology (35).

The prevalence of germline or somatic alterations in DNA repair genes in patients with metastatic prostate cancer has been found to be as high as 20-30% (36, 37). This includes *BRCA1/2*, as well as additional genes such as *ATM, FANCA, CHEK2, PALB2, CDK12*, and *RAD51D*. Due to this high prevalence of mutations, current National Comprehensive Cancer Network (NCCN) guidelines recommend germline testing for homologous recombination repair genes in patients with metastatic prostate cancer (38). In patients for whom one of these mutations is identified, PARPi represent a potential therapeutic strategy in the management of metastatic prostate cancer.

## 2 PARPi monotherapy

### 2.1 Olaparib

Olaparib was the first PARPi extensively studied in the treatment of prostate cancer. It is an orally bioavailable inhibitor of PARP1 and PARP2. In 2015, the phase II TOPARP-A trial studied olaparib in patients with mCRPC enriched for germline and somatic deficiency in DNA repair genes (8). In this study, patients previously treated with taxane-based chemotherapy and hormonal therapy (ADT and androgen receptor signaling inhibitor (ARSI)) received olaparib at a dose of 400mg twice a day. The primary endpoint of the study was response rate, which was defined as either objective response according to Response Evaluation Criteria in Solid Tumors (RECIST), a reduction of 50% or greater in prostate-specific antigen level (PSA_50_), or a confirmed reduction in the circulating tumor-cell count from 5 or more cells per 7.5 ml of blood to less than 5 cells per 7.5 ml. Next generation sequencing (NGS), exome and transcriptome analysis, and polymerase-chain-reaction testing were performed on tumor samples. A response rate of 33% was observed, with a median duration of response of 40 weeks. NGS identified alterations in DNA repair genes in 33% of patients evaluated. Among these patients, 88% demonstrated response to olaparib, including all 7 patients with *BRCA2* loss, whereas among patients without an identified DNA repair defect, the observed response rate was 6%.

Following this study, the TOPARP-B trial selected 98 mCRPC patients who had received prior taxane-based chemotherapy and who had a DNA damage response gene alteration (somatic or germline) identified *via* genetic sequencing (10). Two doses of olaparib (300mg twice daily and 400mg twice daily) were studied. The primary endpoint was response rate using the same criteria to define response as from TOPARP-A. A composite response rate of 54% was observed in the 400mg dose cohort, and 39% for the 300mg dose cohort. The most common adverse event in both dosing cohorts was anemia, and a dose reduction due to any adverse event was required in 37% of patients at the 400mg dose and 12% of patients at the 300mg dose, respectively. The most commonly altered homologous recombination repair gene identified in this study was *BRCA2* (31%), with additional alterations identified in *ATM* (21%), *CDK12* (21%), and *PALB2* (7%). Patients with *BRCA1/2* alterations were found to have the highest response rate (83%) among the alterations identified.

The PROfound study was a phase III trial that evaluated olaparib in men with mCRPC who had disease progression during second generation hormonal therapy (11). Patients additionally were required to have at least one qualifying HRR alteration, and were divided into two cohorts: cohort A, which consisted of patients with *BRCA1, BRCA2*, or *ATM* mutations, or cohort B, which consisted of patients with an alteration in any of 12 other prespecified genes determined from tumor tissue (*BRIP1, BARD1, CDK12, CHEK1, CHEK2, FANCL, PALB2, PPP2R2A, RAD51B, RAD51C, RAD51D*, and *RAD54L*). Patients were randomly assigned in a 2:1 ratio to receive olaparib or the physician’s choice of enzalutamide or abiraterone. The primary end point was radiographic progression free survival (rPFS) in cohort A per blinded independent central review. The study yielded positive results, with a significant increase in rPFS in the olaparib arm as compared to the control arm of cohort A (7.4 months vs 3.6 months, Hazard ratio 0.34, p<0.001). The objective response rate (ORR) was 33% in the olaparib group and 2% in the control group. In the overall population of the study, the median rPFS also favored the olaparib group (5.8 months vs 3.5 months, Hazard ratio 0.49, p<0.001).

Most notably, PROfound was the first study to demonstrate a statistical improvement in overall survival *via* PARPi therapy in prostate cancer. Median overall survival in cohort A was 19.1 months in the olaparib group and 14.7 months in the control group (Hazard ratio 0.69, P=0.02). This survival benefit was seen despite 66% of the control group crossing over to receive olaparib. Additional adverse events in this study included a potential induction of myelodysplastic syndrome (MDS)/acute myeloid leukemia (AML). The results of the PROfound study led to FDA approval in May 2020 for the use of olaparib in patients with mCRPC who have progressed on second generation hormone therapy and who have an identified germline or somatic gene mutation in any homologous recombination repair gene, including *BRCA1/2, ATM, CDK12, CHEK1, CHEK2, PALB2*, or *RAD51D*.

### 2.2 Rucaparib

The phase II TRITON2 study evaluated patients with mCRPC and a germline or somatic *BRCA1* or *BRCA2* alteration who progressed after one or two lines of ARSI therapy and one taxane-based chemotherapy (9). Patients were treated with a starting dose of 600mg rucaparib twice daily. The results of this study showed an overall response rate of 43.5% per independent radiology review and 50.8% per investigator assessment. PSA response rates was 54.8%. Overall response rates were found to be similar for patients with either a germline or somatic *BRCA* alteration, however higher PSA response rate was achieved in patients with a *BRCA2* alteration. Analysis of homologous recombination deficiency gene alterations in non-*BRCA1/2* genes noted minimal response to rucaparib therapy. The most frequent treatment-emergent adverse event noted was anemia, however nausea and elevation in AST/ALT were also common.

Results of the TRITON2 study led to accelerated FDA approval of rucaparib for patients with mCRPC and a germline or somatic *BRCA1/2* alteration who have progressed on ARSI and a taxane-based chemotherapy. This approval was conditional based on the results from the TRITON3 trial, which is a phase III trial of rucaparib vs chemotherapy or second line androgen deprivation therapy in patients with mCRPC with mutations in *BRCA* or *ATM* who had disease progression following treatment with an ARSI (39). This study randomized patients in a 2:1 ratio to receive oral rucaparib 600mg BID or physician’s choice control (docetaxel or ARSI). Primary outcome measured was median rPFS per independent review. Results demonstrated that at 62 months, rPFS was significantly longer in the rucaparib group as compared to the control group in both the *BRCA* subgroup (11.2 months vs 6.4 months; HR 0.50) and in the intention-to-treat group (10.2 months vs 6.4 months; HR 0.61). Exploratory analysis of the *ATM* subgroup noted median rPFS of 8.1 months in the rucaparib group as compared to 6.8 months in the control group. The most common reported adverse events in the rucaparib group were fatigue and nausea. A supplemental new drug application for single agent rucaparib is planned for FDA submission in early 2023 based on these results.

### 2.3 Niraparib

The phase II GALAHAD study evaluated patients with mCRPC enriched for DNA repair gene defects (assessed *via* blood, tumor tissue, or saliva) who had progressed on a prior next-generation androgen signaling inhibitor and a taxane-based chemotherapy (12). DNA repair genes include *BRCA1/2, ATM, FANCA, PALB2, CHEK2, BRIP1*, and *HDAC2*, however mutations must be biallelic per study protocol. Patients were administered niraparib 300mg once daily. The primary endpoint of this study was ORR in patients with *BRCA* alterations and measurable disease. The ORR in the measurable *BRCA* cohort was 34.2%, as compared to 10.6% in the non-*BRCA* cohort. Median duration of response was 5.55 months. In the exploratory endpoint of composite response rate, more than 40% of patients in the BRCA cohort had a PSA_50_ and circulating tumor cell (CTC) conversion, and approximately 2/3 of patients showed a PSA decrease from baseline. Of note, a complete response was observed in 3% (2 of 76) patients in the *BRCA* cohort. The most common adverse events where nausea, vomiting, anemia, and thrombocytopenia.

### 2.4 Talazoparib

The phase II TALAPRO-1 study examined talazoparib in mCRPC patients with HRR alterations (13). The genes examined in this study include *ATM, ATR, BRCA1, BRCA2, CHEK2, FANCA, MLH1, MRE11A, NBN, PALB2* and *RAD51C*. Patients had received prior taxane-based chemotherapy and progressed on enzalutamide, abiraterone, or both. Talazoparib was administered at a dose of 1mg per day or 0.75mg per day in patients with renal impairment. The primary endpoint of this study was confirmed ORR, defined as best overall soft-tissue response per RECIST v1.1 *via* blinded independent central review. ORR was found to be 29.8% (31 of 104 patients), median duration of therapy was 6.1 months. ORR was highest in patients with *BRCA1/*2 mutations as compared to other HRR alterations. The most common adverse events in this study were anemia, thrombocytopenia, and neutropenia.

## 3 PARPi combination strategies

Although the combination of PARPi and underlying homologous recombination repair deficiency appears to be an effective strategy in the management of prostate cancer, ongoing work is being done to determine whether PARPi can be combined with other agents to induce synergistic lethality. These combination strategies aim to expand the population of patients who may benefit from PARPi therapy. To date, PARPi have been combined with androgen deprivation, immunotherapy, cytotoxic chemotherapy, and radiation therapy, with differing results.

### 3.1 PARPi plus ARSI

In the case of androgen deprivation, initial data *in vitro* suggest that androgen deprivation impairs NHEJ and leads to downregulation of DNA repair genes (40, 41). Additional studies have shown that PARP-1 may additionally have a role in supporting androgen receptor (AR) activity, and that inhibition of PARP-1 activity *in vivo* may suppress AR function, decrease tumor growth, and delay the onset of castration resistance (42). Given these data, a phase II study was performed that evaluated the combination of olaparib with abiraterone in patients with mCRPC (14). In this trial, patients were randomly assigned to receive olaparib 300mg twice daily or placebo in combination with abiraterone and prednisone. No selection for mutational status was performed. The primary endpoint was investigator-assessed rPFS. Median rPFS was 13.8 months in the combination olaparib and abiraterone group vs 8.2 months in the placebo and abiraterone group. The most common grade 3 or higher adverse events in the combination group were anemia, pneumonia, and myocardial infarction.

Following this trial, the phase III PROpel trial examined abiraterone plus olaparib vs abiraterone plus placebo in patients with mCRPC in the first line setting (15). Patients were enrolled to this study irrespective of HRR mutation status; however, testing was performed after enrollment to determine HRR status *via* tumor tissue and circulating tumor DNA testing. Patients were assigned to receive abiraterone plus prednisone with either olaparib 300mg twice daily or placebo. The primary endpoint was rPFS *via* investigator assessment. At primary analysis in June 2022, median rPFS was 24.8 months in the abiraterone and olaparib combination arm as compared to 16.6 months in the abiraterone plus placebo arm (hazard ratio 0.66, p<0.001).

Overall survival data, a secondary endpoint, were presented at the GU ASCO 2023 meeting. In the pre-planned final analysis, results showed a consistent trend towards OS benefit in the intention-to-treat population (16). Median OS was found to be 42.1 months in the abiraterone plus olaparib arm as compared to 34.7 months in the abiraterone plus placebo arm. In the subgroup analysis, median overall survival favored the combination of olaparib plus abiraterone over abiraterone plus placebo in all subgroups, with the *BRCA* mutation subgroup showing the greatest amount of benefit. Notably, patients who harbored HRR gene alterations (HRRm), including *BRCA*, were found to have shorter median OS in the placebo group as compared to non-HRR mutated patients (median OS HRRm 28.5 months vs *BRCA*m 23.0 months vs non-HRRm 38.9 months). By contrast, in the combination olaparib plus abiraterone arm, median OS was not reached in the HRRm and BRCAm subgroups at the time of this analysis. The most common adverse events in the combination arm were anemia, fatigue, and nausea.

The phase III MAGNITUDE study is currently ongoing; however, an interim analysis was published at the 2022 Genitourinary ASCO symposium (17). This study is a randomized, double-blind study that examines niraparib vs placebo paired with abiraterone acetate and prednisone as first line therapy in patients with mCRPC with and without HRR gene alterations. Gene alterations in this study include *ATM, BRCA1, BRCA2, BRIP1, CDK12, CHEK2, FANCA, HDAC2*, and *PALB2*. The primary endpoint is rPFS as assessed by blinded independent central review in the *BRCA1/2* group, followed by all HRR biomarker positive patients. Secondary endpoints include time to initiation of cytotoxic chemotherapy, time to symptomatic progression, and overall survival. At the first interim analysis, the combination of niraparib and abiraterone had significantly improved rPFS in the *BRCA1/2* subgroup (Hazard ratio 0.53, p=0.0014), and in all HRR biomarker positive patients (Hazard ratio 0.73, p=0.0217). Analysis in HRR biomarker negative patients did not show any benefit of adding niraparib to abiraterone therapy in this group.

In the second interim analysis of the MAGNITUDE study, secondary endpoints were reported (18). At a median follow up of 26.8 months, a 45% reduction in the risk of progression or death was reported in the niraparib plus abiraterone group as compared to the abiraterone plus placebo group. Updated descriptive rPFS results were consistent with the primary analysis in the HRR+ cohort. In the *BRCA* subgroup, the combination of niraparib and abiraterone therapy extended median rPFS from 10.9 months (abiraterone plus placebo) to 19.5 months. The combination of niraparib and abiraterone additionally led to statistically significant benefit in time to symptomatic progression in the HRR+ cohort. Clinical benefit in time to cytotoxic chemotherapy was also observed in the niraparib plus abiraterone arm in the HRR+ cohort and in the *BRCA* subgroup. Additionally, *BRCA* patients treated with combination therapy experienced delayed time to worst pain intensity (HR 0.70) and pain interference (HR 0.67) as compared to the abiraterone plus placebo group. No new safety signals were observed at the second interim analysis. Overall survival benefit was not conclusive due to immaturity of data at the second interim analysis and will be followed through to the final analysis.

The currently ongoing TALAPRO-2 study is a phase III study that compares talazoparib plus enzalutamide vs placebo plus enzalutamide as first line therapy for patients with mCRPC with or without HRR alterations (43). Prior chemotherapy was allowed in metastatic castration sensitive disease, however no prior ARSI therapy was permitted prior to enrollment. This study has co-primary endpoints of rPFS by blinded independent clinical review in all-comers (cohort 1) and in patients with identified HRR alterations (cohort 2). Secondary endpoints include overall survival, time to toxic chemotherapy, ORR, patient-reported outcomes, and safety. Patients were randomized 1:1 to receive either talazoparib 0.5 mg daily plus enzalutamide or placebo plus enzalutamide.

At a median follow up of approximately 25 months, the primary endpoint of rPFS showed a 37% risk of progression or death in the combination talazoparib and enzalutamide group (44). Median rPFS was not yet reached in the combination therapy arm and was found to be 21.9 months in the enzalutamide plus placebo arm. In the HRR+ subgroup analysis, rPFS was 27.9 months vs 16.4 months in the combination vs control group, respectively. ORR and complete response (CR) rates were higher in the combination arm as compared to control as well (ORR 61.7% vs 43.9% respectively; CR 37.5% vs 18.2%, respectively). The most common treatment related side effects in the talazoparib arm were anemia, neutropenia, and thrombocytopenia. Overall survival data were not yet mature at the time of this follow up. The study concluded that all patients in the study benefitted from the combination of talazoparib and enzalutamide in the first line setting, although the benefit appears to be most pronounced in HRR+ patients.

In addition to this study, the combination of talazoparib and enzalutamide is being studied in the phase III TALAPRO-3 trial, which is investigating this combination in men with HRR-deficient metastatic castration sensitive prostate cancer (mCSPC). In this study, HRR-deficient genes include *ATM, ATR, BRCA1, BRCA2, CDK12, CHEK2, FANCA, MLH1, MRE11A, NBN, PALB2*, and *RAD51C*. The primary endpoint is rPFS, and secondary endpoints include overall survival, safety, and patient reported outcomes.

Lastly, the phase II NCI 9012 trial investigated the combination of abiraterone plus prednisone with or without the PARPi veliparib (19). This study additionally stratified patients by ETS status to investigate whether ETS fusions (e.g. *ERG : TMPRSS2* translocation) predict response to therapy. These fusions were selected for stratification due to prior studies that have shown that ETS fusions are common driving events in prostate cancer. The primary objectives from this study were PSA response rate and whether ETS fusions predicted response. Overall, this was a negative study in that the addition of veliparib and ETS status testing respectively did not affect response. There were no differences in PSA response rate nor in median PFS. Exploratory biomarker analysis comparing HRR alteration positive vs HRR wild type patients did show significantly higher PSA response rates and increased rPFS in both arms of the study in patients who were biomarker positive for HRR alterations.

Altogether, these clinical studies demonstrate a role for PARPi in combination with ARSI. Several trials are currently ongoing that investigate the efficacy of these combinations of therapies (**Table 3**). It appears that outcomes may differ based on selection of PARPi and of ARSI, thus the optimal combination of these therapies may be a subject of future investigations.

**Table 3:** Ongoing trials of PARPi combination therapies in mCRPC.

Trial, NCT#	Phase	PARPi	Combined Agent	Population
NiraRad, NCT03076203	I	Niraparib	Radium-223	mCRPC with osseous metastases
NCT03874884	I	Olaparib	Lutetium-177-PSMA-617	mCRPC, unselected for HRR
NCT04703920	I	Talazoparib	Belinostat	mCRPC, metastatic breast cancer, metastatic ovarian cancer, unselected for HRR
NCT04586335	I	Olaparib	CYH33	HRR+, progressed on one PARPi
COMRADE, NCT03317392	I/II	Olaparib	Radium-223	mCRPC with osseous metastases
NCT05252390	I/II	Olaparib	NUV-868	mCRPC, unselected for HRR
NCT04267939	Ib/II	Niraparib	Elimusterib	Advanced solid tumors
NCT04734730	II	Talazoparib	Abiraterone	mCRPC, unselected for HRR
NCT04019327	II	Talazoparib	Temozolomide	mCRPC, unselected for HRR
NCT05005728	II	Olaparib	XmAb20717	mCRPC, unselected for HRR
NCT03516812	II	Olaparib	Bipolar androgen therapy	mCRPC, unselected for HRR
NCT04824937	II	Talazoparib	Telaglenastat	mCRPC, unselected for HRR
NCT02893917	II	Olaparib	Cediranib	mCRPC, unselected for HRR
NCT04592237	II	Niraparib	Cetrelimab	Aggressive variant mCRPC
CASPAR, NCT04455750	III	Rucaparib	Enzalutamide	mCRPC, unselected for HRR
TALAPRO-2, NCT03395197	III	Talazoparib	Enzalutamide	mCRPC, unselected for HRR
AMPLITUDE, NCT04497844	III	Niraparib	Abiraterone	mCSPC, HRR+

### 3.2 PARPi plus immunotherapy

PARPi in combination with immunotherapy has additionally been investigated. Notably, except for the tissue-agnostic FDA approval for the use of pembrolizumab in tumors that exhibit high tumor mutational burden (TMB) or microsatellite instability, there is no current FDA approval for immune checkpoint inhibitors (ICI) in the management of prostate cancer. However, emerging trends in ICI response suggest that there may be some populations of patients who may benefit from the incorporation of ICI into treatment regimens. The basis for further exploration into the combination of PARPi and ICI is derived from multiple studies that suggest that PARPi-mediated DNA damage may modulate the tumor immune microenvironment (45).

Results from studies of tumors with high TMB have suggested that TMB is a surrogate of neoantigen load and may predict therapeutic response to ICI (46–48). Additional data show that there may be a correlation between high TMB and HRR deficiency (49). Thus, the combination of PARPi and ICI represents a reasonable strategy for targeting response in patients with HRR deficiency. In addition, by impacting DNA repair *via* PARPi, researchers have theorized that the resultant DNA damage may increase neoantigen load and thusly TMB, therefore potentially making tumors more susceptible to ICI therapy.

In a phase II study, patients with mCRPC with and without somatic or germline HRR alterations were treated with durvalumab 1500mg IV every 28 days and olaparib 300mg PO twice daily (20). Previous treatment with an ARSI therapy was required, but prior chemotherapy was not required. Median rPFS for all patients was 16.1 months with a 12-month rPFS of 51.5%. In the subset of patients with HRR alterations, median rPFS was 16.1 months. Nine of 17 (53%) patients had a radiographic and/or PSA_50_ response. Among the study patients, 4 of 17 had immune-related adverse events, however no patients were taken off trial for toxicity. PD-L1 expression did not appear to be correlated with response.

In the CheckMate 9KD study, a multi-cohort phase II trial was conducted of patients with mCRPC (21). In the A2 cohort, patients with mCRPC who had received prior abiraterone or enzalutamide therapy but not chemotherapy were administered nivolumab 480mg IV every 4 weeks plus rucaparib 600mg PO twice daily. Coprimary endpoints were ORR and PSA response rate in all treated patients and in patients with HRR alterations. HRR status was determined prior to enrollment. In a total of 71 patients, 34 patients were found to be HRR alteration positive, and 37 patients were HRR negative. Overall, the ORR was 39% and 66% of patients achieved a PSA_50_. Median rPFS was 8.1 months for all patients but was 10.9 months for HRR+ patients vs 5.6 months in HRR- patients. In the A1 cohort of this study, patients with mCRPC were more heavily pre-treated and had received prior abiraterone or enzalutamide and prior chemotherapy. These patients were similarly treated with nivolumab plus rucaparib (22). In this cohort, 88 patients were treated; 45 patients were HRR+ and 43 patients were HRR-. The ORR was 10.3% overall, and 17.2% in HRR+ patients vs 3.4% in HRR-. Additionally, the confirmed PSA_50_ was 11.9% overall, 18.2% in HRR+, and 5.0% in HRR-. In patients with *BRCA2* mutations, confirmed ORR was 37.5% (3 of 8 patients) and confirmed PSA_50_ was 45.5% (5 of 11 patients). Overall median rPFS was 4.9 months and median OS was 13.9 months. Most common adverse events included anemia, neutropenia, nausea, and fatigue.

The KEYNOTE-365 study is a phase Ib/II study evaluating pembrolizumab plus other agents in mCRPC patients who had previously received docetaxel chemotherapy. HRR alterations were not required for enrollment, and it should be noted that there were challenges with assessment of HRR status in this study due to issues with the ctDNA assay used in the early part of this trial. In cohort A, patients are administered pembrolizumab 200mg IV every 3 weeks plus olaparib 400mg PO twice daily (23). The primary endpoints in this study are safety, PSA_50_, and ORR per blinded independent central review. Of 102 treated patients, 29% were noted to be PD-L1 positive. PSA_50_ was 15%, ORR was 8.5%, and the disease control rate (DCR) was 26%. Median rPFS was 4.5 months and median OS was 14 months. Immune-mediated adverse events occurred in 12 patients (12%), with approximately 4% experiencing a grade 3-5 toxicity.

Based on the results of the KEYNOTE-365 study, the phase III KEYLYNK-010 study combining pembrolizumab plus olaparib was launched (50). In this study, patients with mCRPC unselected for HRR status who had received prior taxane-based chemotherapy and one ARSI were eligible for inclusion. The study design compared pembrolizumab plus olaparib versus abiraterone or enzalutamide. The study was terminated after a preplanned futility analysis as no benefit from the combination of pembrolizumab and olaparib was demonstrated at the time of interim analysis.

The combination of talazoparib and avelumab is currently being studied in the JAVELIN PARP Medley trial (24). This is a phase Ib/II basket trial of patients with advanced solid tumors including mCRPC both with and without HHR alterations. Patients were administered avelumab 800mg every 2 weeks plus talazoparib 1mg once daily. In the phase 2 trial mCRPC cohort, no confirmed ORs were reported, however PSA responses were observed in 2 of 21 patients. In the HHR positive mCRPC cohort, the ORR was 11.1%. These data are being used for the basis for future clinical trial generation.

### 3.3 PARPi plus chemotherapy

PARPi have been investigated in combination with cytotoxic chemotherapy with the notion that damage induced by chemotherapy may then facilitate synthetic lethality *via* PARPi. In a small 25-patient single-arm study of patients with mCRPC, the combination of veliparib with temozolomide was studied (25). Patients who had progressed on at least one docetaxel-based chemotherapy were eligible. Patients were treated with veliparib 40mg twice daily on days 1-7 and temozolomide daily on days 1-5 of a 28-day cycle. The results of the trial demonstrated that this combination was tolerated but had only modest activity. No objective responses were observed and only 2 of 25 patients had confirmed PSA decline of 30% or greater. HRR status was not assessed. This lack of activity might be due to lack of HRR status selection, the selection of PARPi as veliparib has comparatively less activity than other PARPi, or due to selection of chemotherapy as temozolomide is not commonly used as a cytotoxic agent in the management of prostate cancer. A trial of talazoparib plus temozolomide for patients with mCRPC is currently actively recruiting (NCT04019327).

Additional combination chemotherapy and PARPi studies are uncommon, largely due to concern for increased toxicity, however the role of PARPi as maintenance after cytotoxic therapy is currently under investigation. Two studies are currently ongoing to address this question (NCT03442556 and NCT03263650).

### 3.4 PARPi plus radiotherapy

Radium-223 is a targeted alpha-particle therapy that is currently utilized in the management of metastatic prostate cancer with symptomatic osseous disease (51). This form of therapy causes damage *via* DNA double-strand breaks. Prior *in vivo* studies have shown that PARPi may act as a radiosensitizing agent, thereby increasing the efficacy of radiation therapy (52).

The proposed synergistic combination of radium-223 and olaparib is currently being investigated in the phase I/II COMRADE trial (53). Initial phase I data published noted that the recommended dose of olaparib was reduced to 200mg twice daily as compared to the typical dosing used in monotherapy due to anemia and thrombocytopenia observed at higher doses (54). Updated phase I results from this study reported that of 12 patients enrolled, all patients had received prior ARSI therapy, and 3 patients received prior docetaxel (55). Dose limiting toxicities included cytopenias, fatigue, and nausea. rPFS at 5 months was 58% (95% CI, 27%-80%). Among nine patients who were evaluable for HRR gene status, 1 had a *BRCA2* alteration (rPFS 11.8 months), and 1 had a *CDK12* alteration (rPFS 3.1 months). The ongoing phase II study randomizes patients to radium-223 alone vs the combination of radium-223 and olaparib. The primary endpoint is rPFS, and HRR status will be documented for analysis.

A phase Ib study of niraparib in combination with radium-223 has also been performed (56). The combination of these two therapies was shown to have adequate safety, with the most common adverse events being anemia and neutropenia. A secondary analysis of PSA_50_ response was 10% and was 14-30% (dose-dependent) in the chemotherapy-naïve subgroup.

Recently FDA approved, Lutetium-177-PSMA-617 is a beta-emitter therapy that is conjugated with a small molecule that targets PSMA (57). An early phase study of patients with mCRPC who have received prior chemotherapy and ARSI therapy combining olaparib with Lutetium-177-PSMA-617 is ongoing (NCT03874884).

### 3.5 PARPi plus targeted therapy

Targeted therapy presents a potential mechanism by which to target DNA repair pathways in addition to PARP inhibition to induce lethality in cancer cells. In a randomized control trial of olaparib with or without the vascular endothelial growth factor (VEGF) receptor inhibitor cediranib, patients with progressive mCRPC were randomly assigned to receive either cediranib 30mg once daily plus olaparib 200mg twice daily or olaparib 300mg twice daily alone (58). Based on preclinical models, the combination of VEGF inhibitor with PARPi was hypothesized to increase sensitivity to PARPi. This study found that in the intention-to-treat set of patients, median rPFS was 8.5 months in the combination arm vs 4.0 months in the PARPi monotherapy arm. The combination of cediranib and olaparib significantly improved rPFS as compared to olaparib alone (HR 0.617; CI 0.392-0.969, p=0.0359). Among patients with HRR-deficient mCRPC, a median rPFS of 10.6 months vs 3.8 months was observed in the combination vs monotherapy arms. In the subset of patients with *BRCA2*-mutated CRPC, median rPFS was 13.8 months in the combination arm vs 11.3 months in the olaparib only arm. Notably, the combination arm had a higher incidence of grade 3-4 adverse events as compared to the monotherapy arm (61% vs 18% respectively).

In an *in vitro* study of olaparib and the ATR inhibitor ceralasterib, this therapeutic combination was shown to selectively cause cell death in *ATM*-deficient cells (59). This synergistic interaction was used as the basis for the TRAP trial, a 2-cohort study of patients with *BRCA1*, *BRCA2*, or *ATM* mutations or without any HRR alterations (26). In this study, olaparib was administered twice daily at a standard dose, and ceralasterib was administered daily on days 1-7 of a 28-day cycle. The primary endpoint was disease response (confirmed PSA_50_ or RECIST response). Response rate in the HRR cohort was 33% vs 11% in the HRR negative cohort.

Additional targeted therapy combination studies include combination studies with PARPi and glutaminase inhibitor telaglenastat (NCT04824937), histone deacetylase inhibitor belinostat (NCT04703920), PI3K inhibitor CYH33 (NCT04586335), BET/bromodomain inhibitor NUV-868 (NCT05252390), and ATR inhibitor elimusterib (NCT04267939).

### 3.6 PARPi plus bipolar androgen therapy

Bipolar androgen therapy (BAT) is a strategy that has shown potential benefit in the metastatic castration resistant setting. BAT results in rapid fluctuation of testosterone between near-castrate and supraphysiologic levels and may induce DNA damage and antitumor effect (60, 61). Preclinical studies have suggested synergy between BAT and PARPi, prompting further study into this strategy (62). In a phase II trial testing BAT plus olaparib, the primary objective was to determine PSA_50_ response at 12 weeks (63). 36 patients were enrolled, and in the intention-to-treat cohort, PSA_50_ response rate at 12 weeks was 11/36 (31%; 95% CI 17%-48%), and 16/36 (44%, 95%CI 28%-62%) had a PSA_50_ response at any time point while on study. At a median follow up of 19 months, the median rPFS was 13.0 months. Clinical benefit was observed independent of HRR gene mutational status.

## 4 Differential response among HRR alterations

Review of data from the various PARPi clinical trials is notable for a theme of variable response to PARPi therapy with respect to which gene in the DNA damage repair pathway is mutated. This differential response to PARPi therapy is highlighted in the findings of the PROfound trial, in which rPFS was noted to be markedly increased in the subset of patients with *BRCA2* alterations in particular (11). This distinction is important as historically, many clinicians have considered *BRCA1* and *BRCA2* mutations to behave similarly with respect to therapeutic outcomes. One study investigated this difference in outcomes in metastatic prostate cancer treated with PARPi and found that the efficacy of PARPi is diminished in *BRCA1* altered mCRPC as compared to *BRCA2* (64). The observed difference was proposed to be related to more monoallelic mutations or concurrent *TP53* alterations in the *BRCA1* group rather than an imbalance in germline mutations. Notably, another study found that the discrepancy in PARPi sensitivity between *BRCA1* and *BRCA2* patients appears to be a class effect from PARPi rather than limited to rucaparib (65). Such findings are the basis from which trials have begun to distinguish biallelic mutations as well as genomic co-alterations in biomarker testing (e.g GALAHAD).

Additionally, in the TRITON2 study, differing outcomes can be seen between patients with *BRCA1* and *BRCA2* alterations as compared to those with *ATM* and *CDK12* alterations (9). Even among patients with *BRCA2* alterations in this study, not all patients were found to respond to rucaparib therapy. An observational study was also conducted that reviewed patients with mCRPC and pathogenic germline or somatic mutations in *BRCA1/*2 and *ATM* and noted that PSA_50_ responses to olaparib were achieved in 76% (13/17) of men with *BRCA1/*2 alterations versus 0% (0/6) men with *ATM* mutations (66). Thus, men with mCRPC harboring *ATM* mutations fared worse than their counterparts with *BRCA1/2* mutations in response to PARPi. This suggests that additional understanding regarding HRR gene testing is needed, and biomarker development may help improve therapy selection for subgroups of HRR deficient tumors (67). Given that the benefit from PARPi is not uniform amongst the different HRR pathway genes, consideration should be given when selecting optimal candidates for PARPi therapy.

## 5 Mechanisms of resistance

One of the challenges with PARPi therapy is the limited duration of response to therapy. Although response rates in the setting of DNA damage repair alterations, namely *BRCA1/2*, are encouraging in clinical trials, unfortunately median response time is still on the order of months. For instance, in the two patients with a complete response in the GALAHAD study, total duration of response was less than 10 months (12). Extensive preclinical studies to date have identified several mechanisms of acquired resistance.

Numerous mechanisms result in the reactivation of homologous recombination repair function, thereby compromising the synthetic lethality of PARPi. This reactivation may occur *via* secondary reversion mutations in key HRR genes, including *BRCA1/2*, *RAD51C/D*, and *PALB2* (44, 68). The loss of p53 binding protein 1 is also associated with PARPi resistance as the somatic loss of this protein leads to partial restoration of homologous recombination (69). Mutations in DNA-binding domains of PARP1 and mechanisms that increase PARylation of PARP1 both impact PAR chain activity, and PAR trapping, and thereby can lead to PARPi resistance (70, 71). Additionally, mouse models of BRCA1-deficient mice with mammary tumors were shown to acquire resistance to olaparib *via* activation of the P-glycoprotein drug efflux transporter, thereby reducing cellular availability of PARPi (72). Lastly, protection of the replication fork and stabilization of stalled forks may lead to PARPi resistance (73). PARPi has been shown to mediate fork degradation *via* PTIP and EZH2 proteins, thus loss of these proteins leads to protection of the replication fork from destruction *via* nucleases and confers a pathway of resistance to PARPi therapy (74, 75). Research is currently ongoing that investigates methods to overcome required resistance. As the development of new therapies is in process, the combination therapy strategies mentioned above reflect a mechanism by which to overcome acquired resistance.

## 6 Future directions

PARP inhibitor therapy is an emerging strategy in the management of prostate cancer. To date, the FDA has approved the use of rucaparib in patients with prostate cancer with *BRCA1/2* alterations who have received prior ARSI therapy and taxane-based chemotherapy. Olaparib has additionally been approved by the FDA for a broader set of HRR genes and does not require the receipt of prior taxane-based chemotherapy. We anticipate future approvals for PARPi therapy over the next few years as further investigations elevate the role of PARPi in the treatment of prostate cancer. Future studies may lead to the transition of PARPi to earlier disease states and additionally may support the use of combination strategies with PARPi.

Although resistance to PARPi continues to be a concern, combination strategies represent one mechanism by which resistance may be circumvented or delayed. Further, combination therapies present a strategy by which to incorporate PARPi in the management of patients without underlying HRR alterations so that these patients may also benefit from this therapy. The optimal combination strategy and timing of therapy implementation in the treatment of prostate cancer remains to be elucidated.

As the landscape of prostate cancer therapy continues to evolve, the need for improved biomarkers to better guide selection of therapies has emerged. Decisions regarding PARP inhibitor therapy are challenging, as a better understanding is needed when examining the benefit of combination therapies that utilize PARP inhibition. Many trials to date suggest that the benefit of PARP inhibitor therapy is greater for patients with HRR alterations, especially with alterations in *BRCA*. Although some studies, such as the TALAPRO-2 trial, show benefit for all patients with combination strategies, the extent of benefit between HRR+ and HRR- patients is still unequal. As second-generation hormone therapies continue to be incorporated into earlier stages of treatment, the degree of benefit from later addition of a PARP inhibitor remains a subject of study. Improved identification of biomarkers to predict response from PARPi therapy will better help weigh the risks of side effects with the potential benefit of PARPi strategies. Understanding the optimal genomic alterations will aid in appropriate patient selection to derive maximal benefit from PARPi.

## Author contributions

All authors contributed to the writing and editing of the manuscript.
